# Supplementary material for: Advancing towards cancer theragnostic by probing the 225Ac decay chain with ultra-high-resolution metallic magnetic calorimeter based detectors
Source: Commun Med (Lond). 2026 Mar 27;6:169. doi: 10.1038/s43856-026-01377-0 (PMC13031560; doi:10.1038/s43856-026-01377-0)
Supplement: Supplementary file 2 — Supplementary information [file 43856_2026_1377_MOESM2_ESM.pdf]

Advancing towards cancer theragnostic by probing the ^{225}Ac decay chain with ultra-high-resolution metallic magnetic calorimeter based detectors

Supporting Information

Maurer, Kiara[1‡]; Unger, Daniel[2‡]; Behe, Martin[3]; Knecht, Andreas[3]; von Schoeler, Katharina[4]; Hengstler, Daniel[2]; Vitova, Tonya[1]; Benešová-Schäfer, Martina[5]; Fleischmann, Andreas[2*]; Gastaldo, Loredana[2]; Wängler, Carmen[6*]; Enss, Christian[2,7*]; Schacherl, Bianca[1*]

[1] Karlsruhe Institute of Technology (KIT), Institute for Nuclear Waste Disposal (INE), P.O. Box 3640, 76021, Karlsruhe, Germany
[2] Kirchhoff Institute for Physics, Heidelberg University, Im Neuenheimer Feld 227, 69120 Heidelberg, Germany
[3] Paul Scherrer Institute (PSI), Forschungsstraße 111, 5232 Villigen, Schwitzerland
[4] ETH Zürich, Institute for Particle Physics and Astrophysics, Otto-Stern-Weg 5, 8093 Zürich, Switzerland
[5] German Cancer Research Center (DKFZ), Research Group Translational Radiotheranostics, Im Neuenheimer Feld 223, 69120 Heidelberg, Germany
[6] Medical Faculty Mannheim of Heidelberg University, Clinic of Radiology and Nuclear Medicine, Biomedical Chemistry, Theodor-Kutzer-Ufer 1-3, 68167 Mannheim, Germany
[7]Karlsruhe Institute of Technology (KIT), Institute for Electronics and Data Processing (IPE), Hermann-von-Helmholtz-Platz 1, D-76344 Eggenstein-Leopoldshafen, Germany

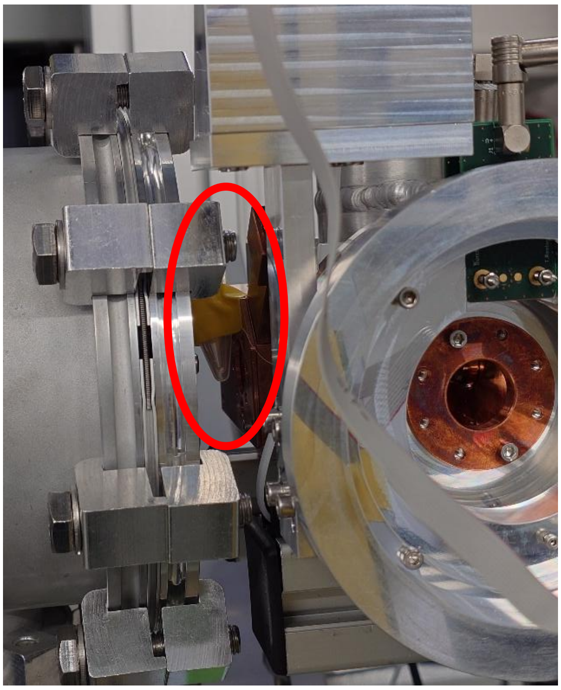

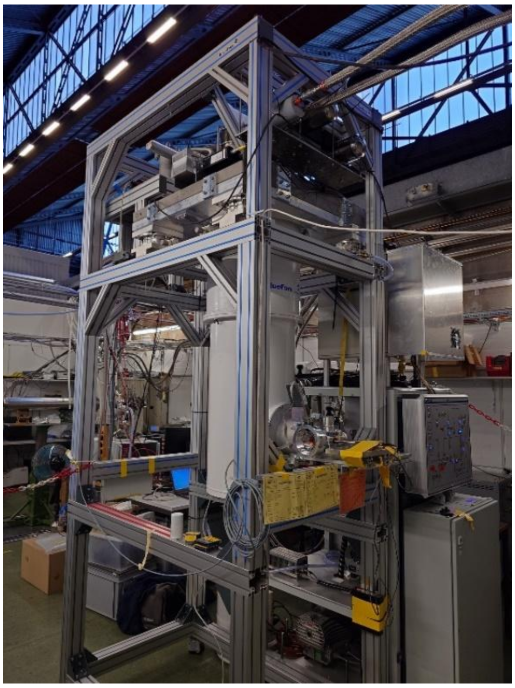

Figure S1: Set-up detection of ultra high-resolution X-ray and γ-spectroscopy of ^{225}Ac (89 keV). The ^{225}Ac sample was filled inside a doubly contained Eppendorf tube (red circled) and placed approximately 8 cm in front of the MMC detectors.

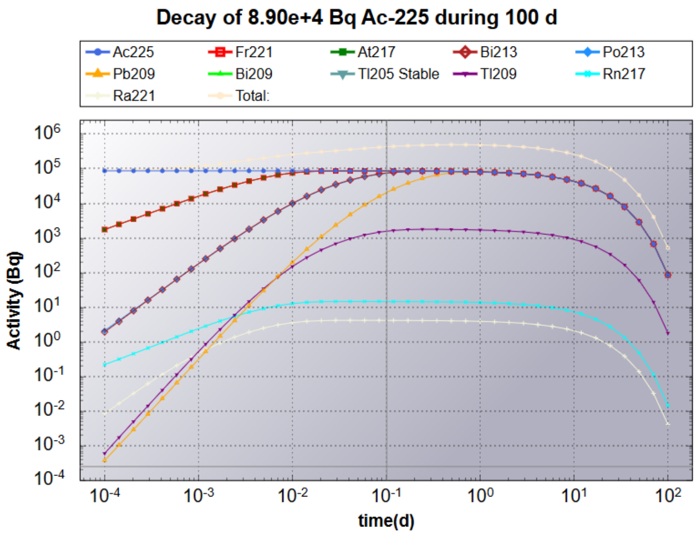

Figure S2: Activity profile of ^{225}Ac and its daughter nuclides[1] (100 days).

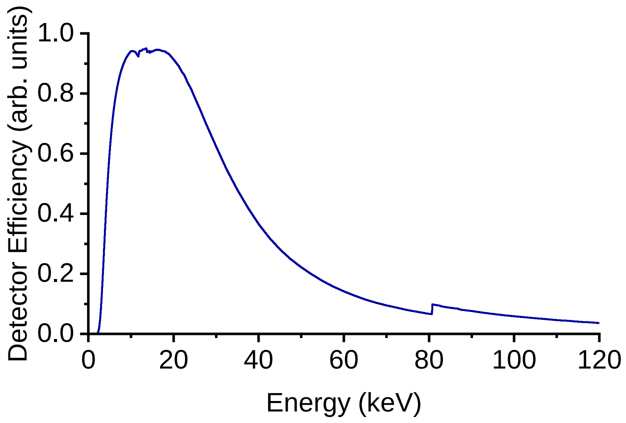

Figure S3: MMC-based detector efficiency from 0 to 125 keV.

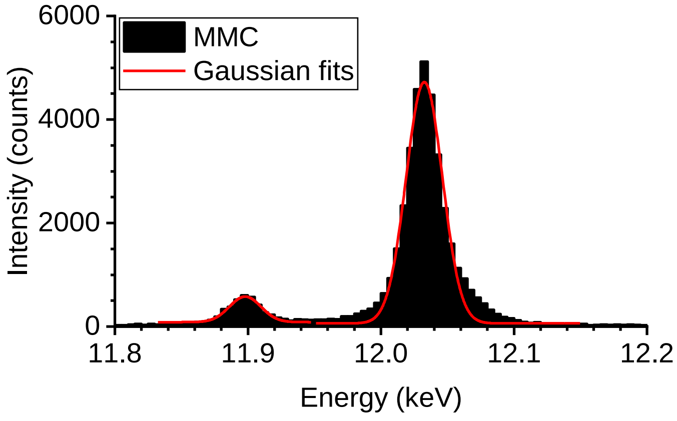

Figure S4: Two intensity signals within < 0.2 keV: 11.90 keV and 12.03 keV.

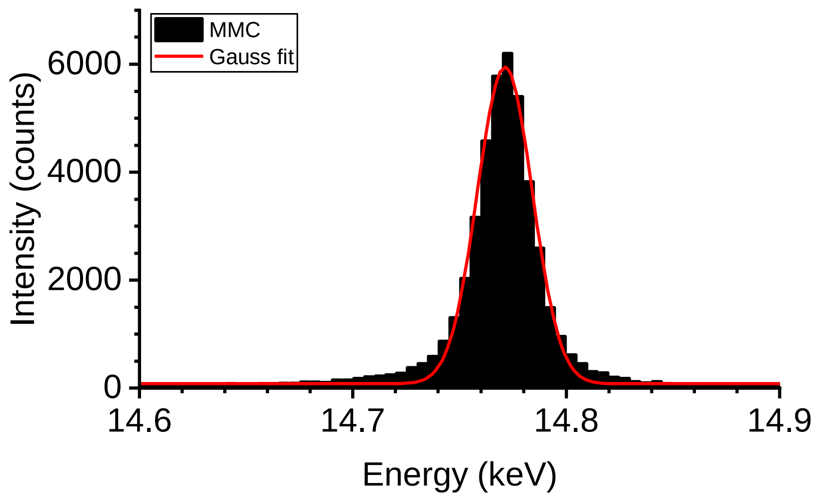

Figure S5: One intensity peak with FWHM 0.03 ± 4.73E-4 keV @ 14.77 keV (binsize: 5 eV).

Experimental MMC spectrum plotted on different energy regions:

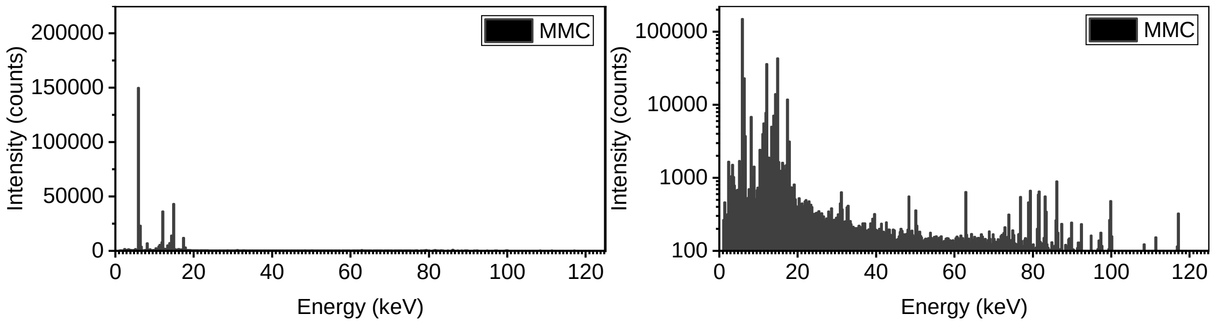

Figure S6: Overview experimental MMC spectrum from 0 to 125 keV (left) Intensity in logarithmic scale and (right) Intensity in linear scale (binsize: 240 keV).

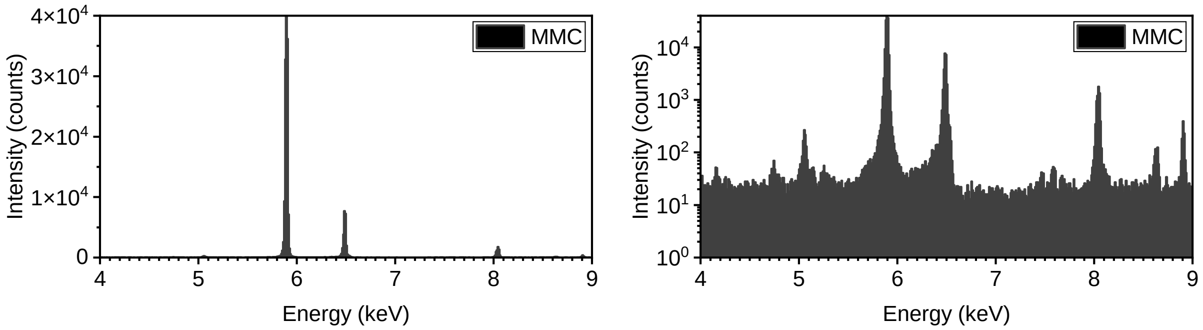

Figure S7: Experimental MMC spectrum of ^{225}Ac from 4 to 9 keV (left) Intensity in logarithmic scale and (right) Intensity in linear scale (binsize: 10 eV).

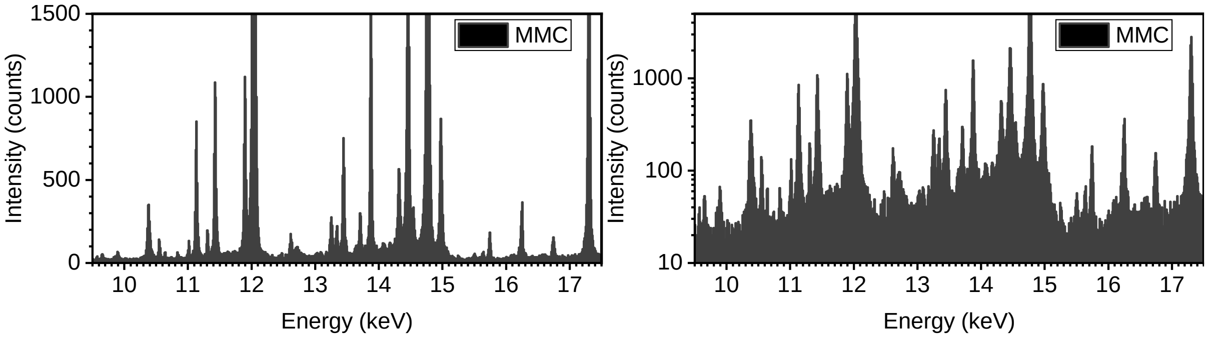

Figure S8: Experimental MMC spectrum of ^{225}Ac from 9.5 to 17.5 keV (left) Intensity in logarithmic scale and (right) Intensity in linear scale (binsize: 10 eV).

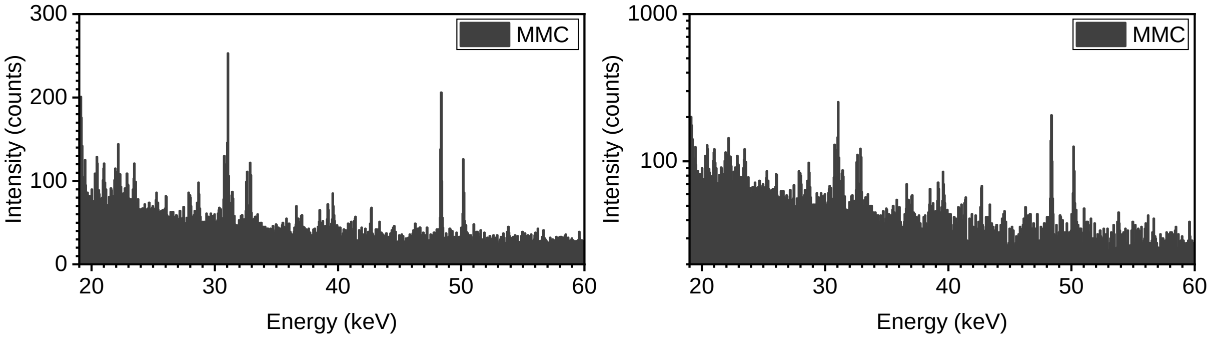

Figure S9: Experimental MMC spectrum of ^{225}Ac from 19 to 60 keV (left) Intensity in logarithmic scale and (right) Intensity in linear scale (binsize: 50 eV).

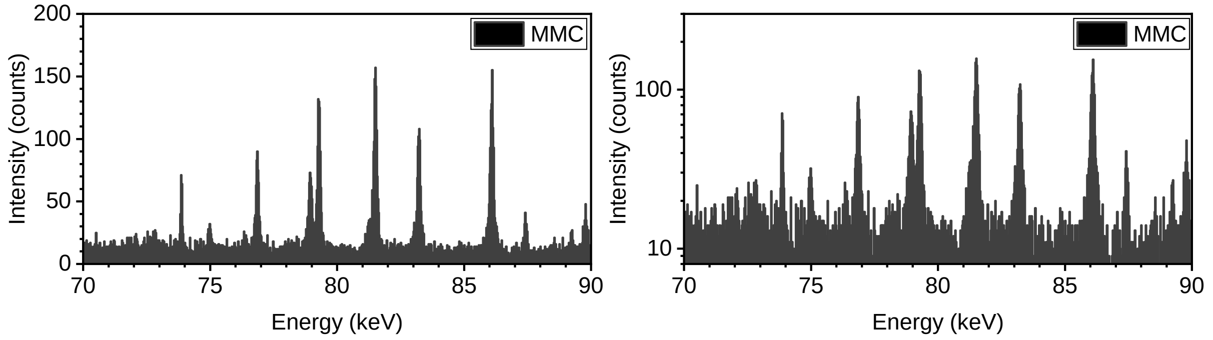

Figure S10: Experimental MMC spectrum of ^{225}Ac from 70 to 90 keV (left) Intensity in logarithmic scale and (right) Intensity in linear scale (binsize: 25 eV).

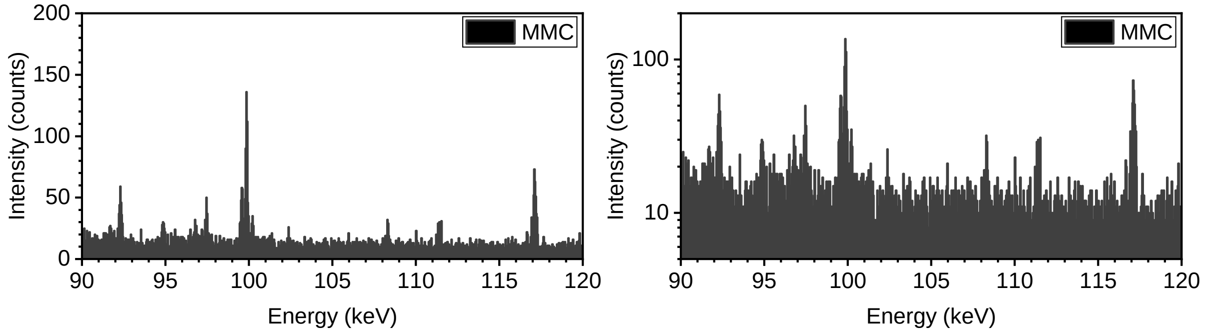

Figure S11: Experimental MMC spectrum of ^{225}Ac from 90 to 120 keV (left) Intensity in logarithmic scale and (right) Intensity in linear scale (binsize: 40 eV).

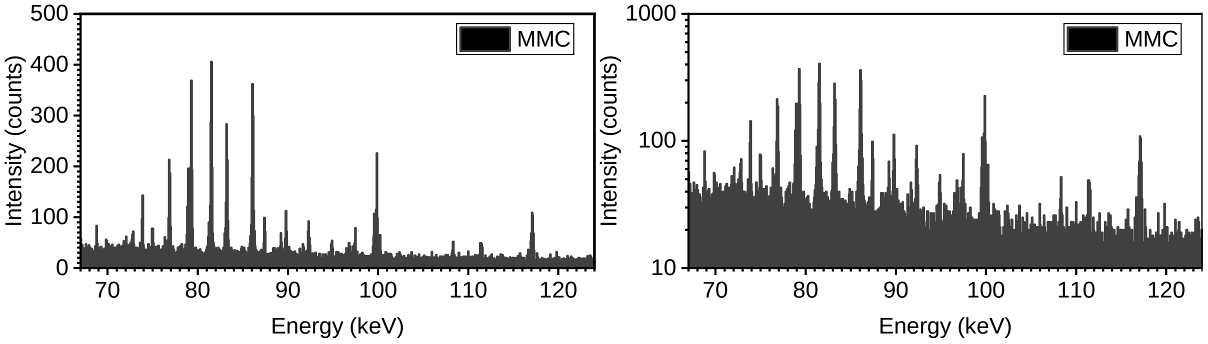

Figure S12: Experimental MMC spectrum of ^{225}Ac from 65 to 125 keV (left) Intensity in logarithmic scale and (right) Intensity in linear scale (binsize: 70 eV).

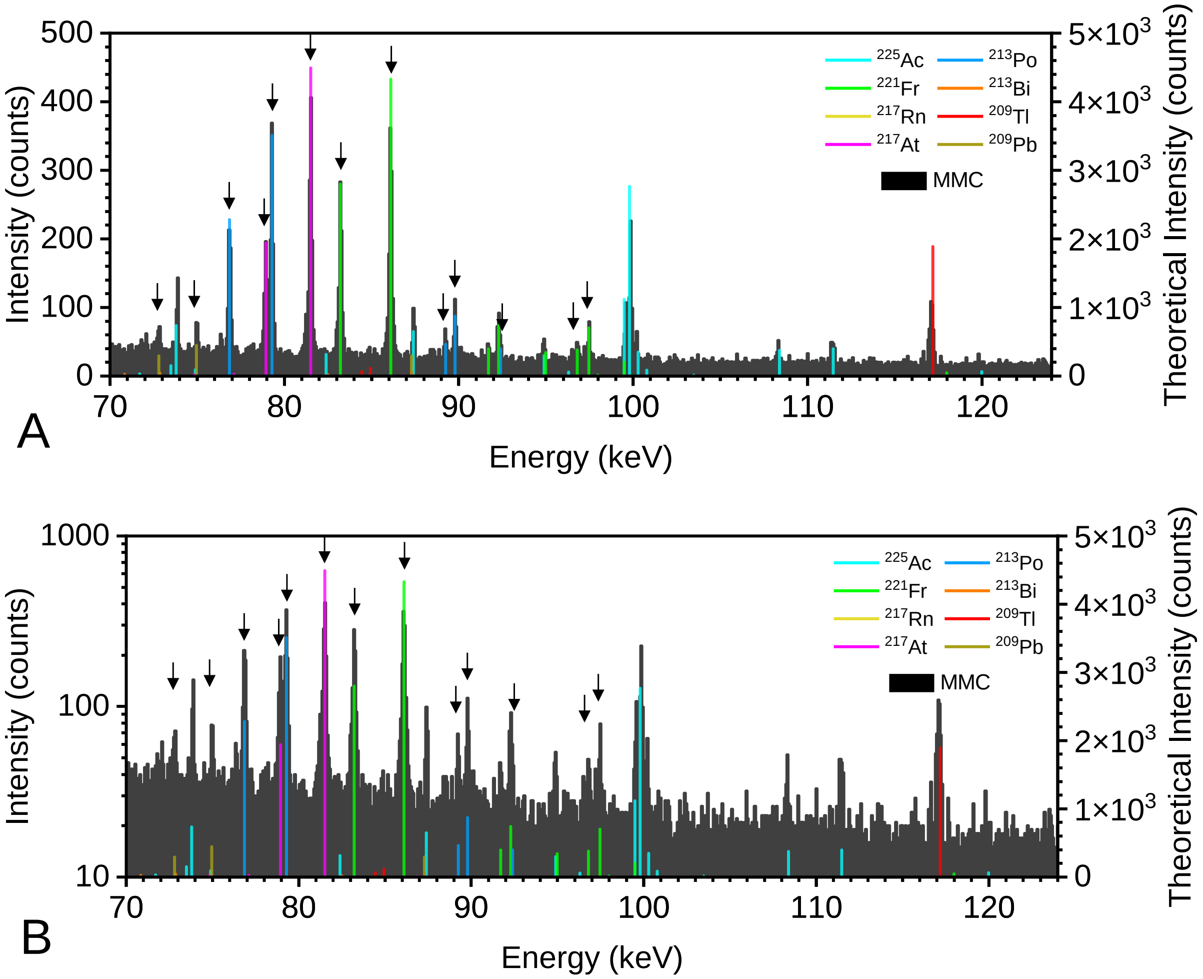

Figure S13: Recorded γ-spectra of ^{225}Ac (black, left y-axis) and theoretically calculated γ-spectra of ^{225}Ac (Nucleonica[1]) efficiency of MMC detectors taken into account (colored, right y-axis) (binsize: 70 eV) (A: intensity linear scale; B: intensity logarithmic scale). The K_α-X-rays of the respective isotopes are marked with arrows.

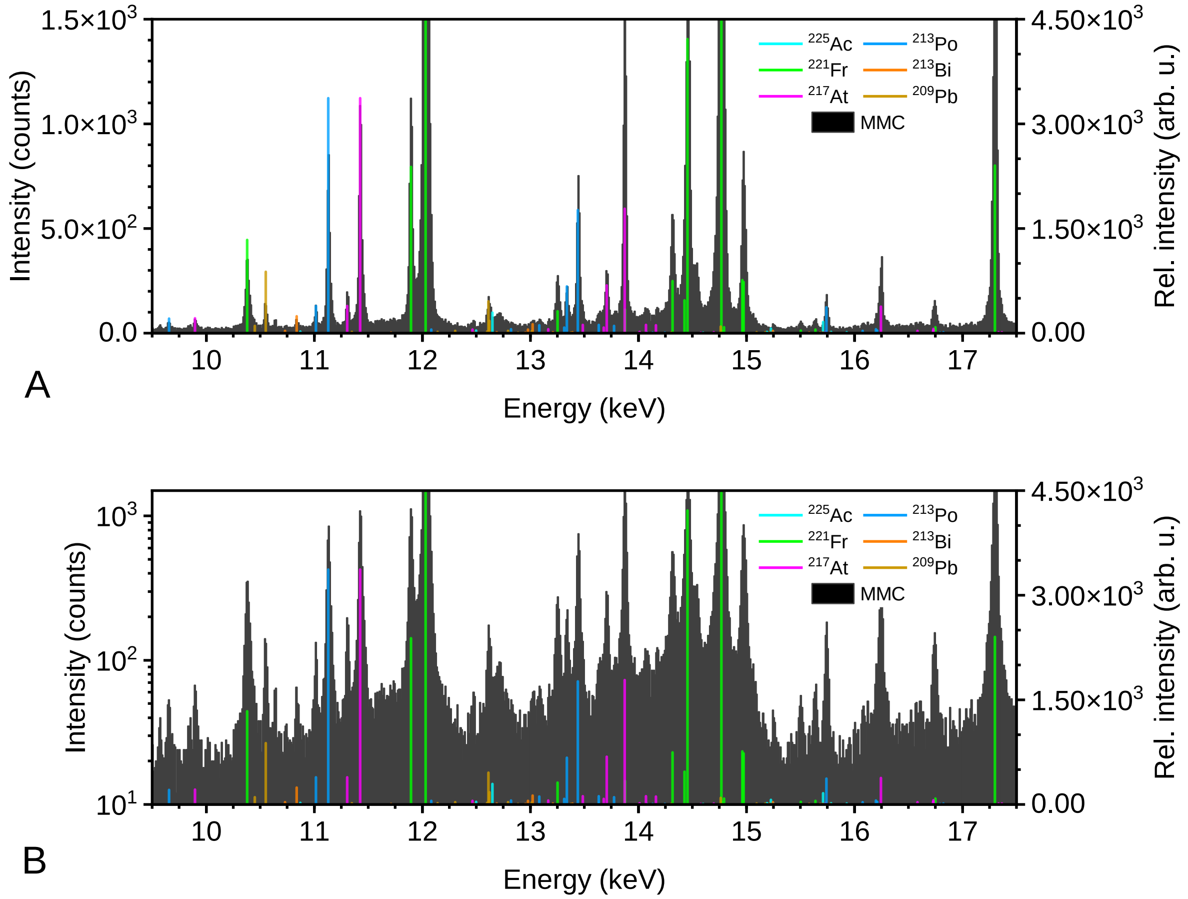

Figure S14: Recorded spectra of ^{225}Ac ((MMC) black, left y-axis) and theoretical X-rays of ^{225}Ac and its daughters (colored, right y-axis (Hephaestus and PyMca[2, 3]) (binsize: 10 eV) (A: intensity linear scale; B: intensity logarithmic scale).

Transmission of photon energy through different materials with different densities

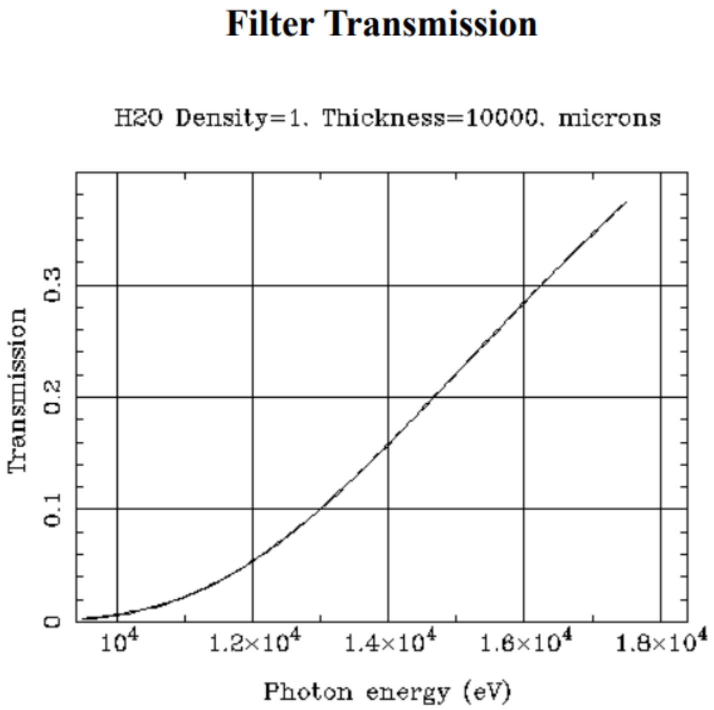

Figure S15: Transmission of photon energy (9.5-17.5 keV) through water (density 1 g/cm^3; thickness 1 cm).

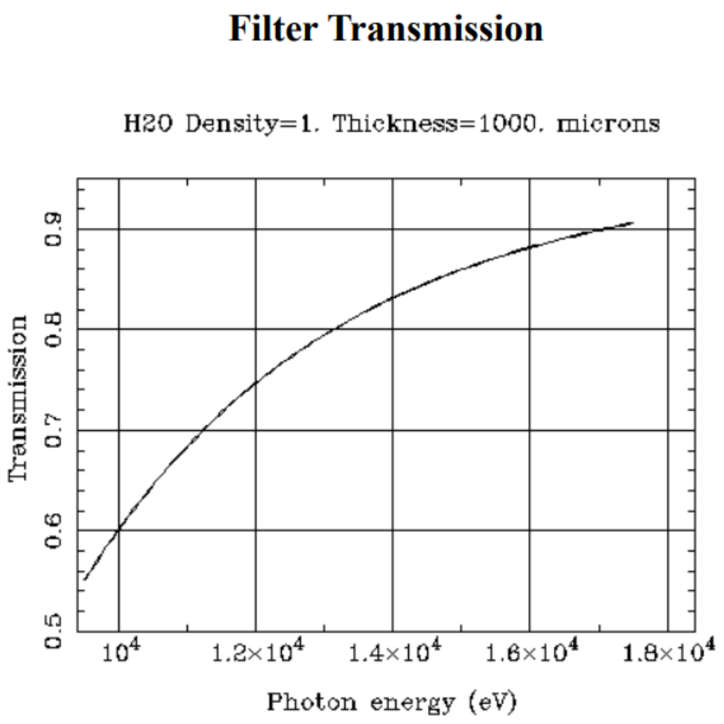

Figure S16: Transmission of photon energy (9.5-17.5 keV) through water (density 1 g/cm^3; thickness 1 mm).

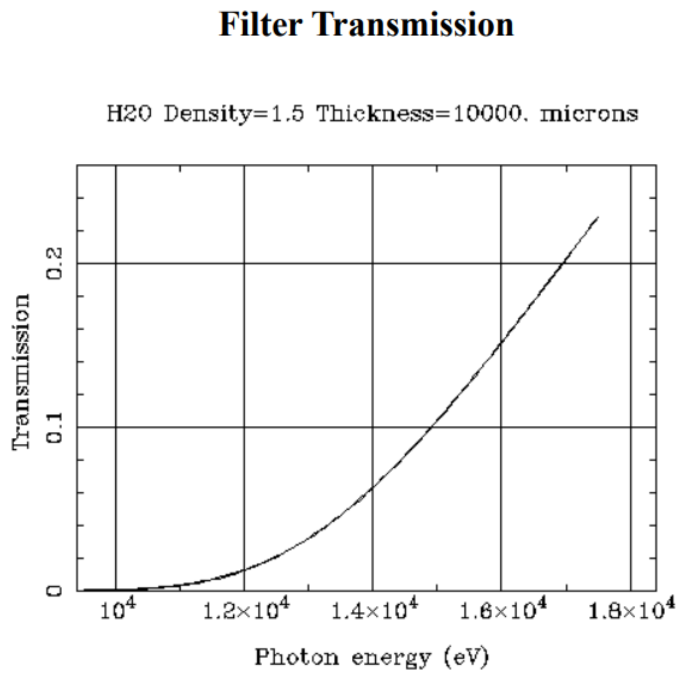

Figure S17: Transmission of photon energy (9.5-17.5 keV) through water (density 1.5 g/cm^3; thickness 1 cm).

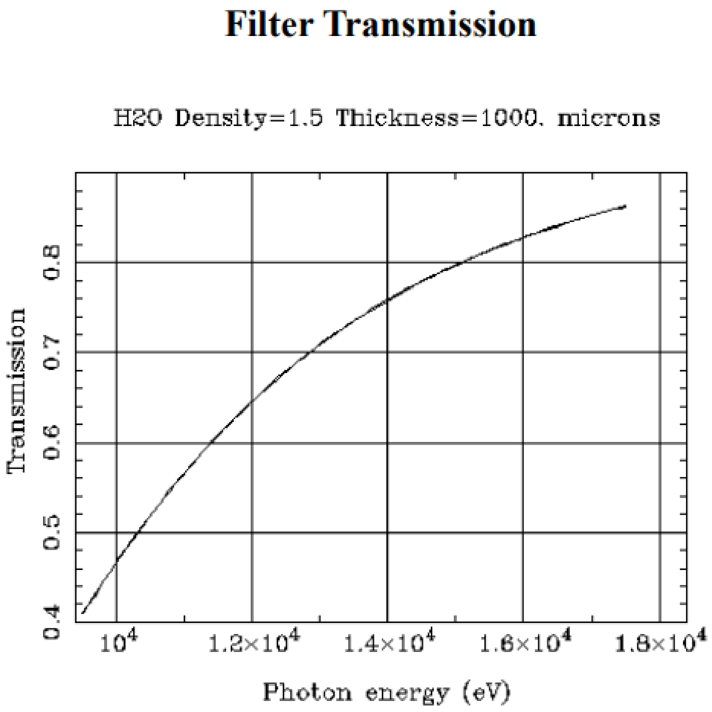

Figure S18: Transmission of photon energy (9.5-17.5 keV) through water (density 1.5 g/cm³; thickness 1 mm).

Supplementary References

(1) Magill, J.; Galy, J.; Dreher, R.; Hamilton, D.; Tufan, M.; Normand, C.; Schwenk-Ferrero, A.; Wiese, H. W. NUCLEONICA: a nuclear science portal.

(2) Ravel, B.; Newville, M. ATHENA, ARTEMIS, HEPHAESTUS: data analysis for X-ray absorption spectroscopy using IFEFFIT. *Journal of Synchrotron Radiation* **2005**, *12* (4), 537-541. DOI: 10.1107/S0909049505012719.

(3) Solé, V. A.; Papillon, E.; Cotte, M.; Ph, W.; Susini, J. A multiplatform code for the analysis of energy-dispersive X-ray fluorescence spectra. *Spectrochimica Acta Part B: Atomic Spectroscopy* **2007**, *62* (1), 63-68. DOI: 10.1016/j.sab.2006.12.002.
